# Risk Factors for Chronic Non-Communicable Diseases of Long-Haul Truck Drivers during the COVID-19 Pandemic: An Integrative Review

**DOI:** 10.3390/ijerph21070897

**Published:** 2024-07-09

**Authors:** Fernanda Lise, Mona Shattell, Flávia Lise Garcia, Laurel Kincl

**Affiliations:** 1 Nursing Faculty, Federal University of Pelotas, Pelotas 96010-610, RS, Brazil; 2 College of Nursing, University of Central Florida, Orlando, FL 32826, USA; mona.shattell@ucf.edu; 3 Anthropology Faculty, Federal University of Pelotas, Pelotas 96010-770, RS, Brazil; flavialisegarcia@gmail.com; 4 College of Health, Oregon State University, Corvallis, OR 97331, USA; laurel.kincl@oregonstate.edu

**Keywords:** social determinants of health, work–life balance, COVID-19, health, work-environmental, working conditions, truckers

## Abstract

Long-haul truck drivers are responsible for transporting goods valued at millions of dollars of the world economy, and may have their health affected by living and working conditions. This study analyzed and synthesized scientific findings about risk factors for the development of chronic non-communicable diseases in long-haul truck drivers. An integrative literature review was conducted. We identified 23 studies that met the inclusion criteria and evaluated the health of 7363 drivers. The biological risk factors identified were age, gender, race/ethnicity, genetics, and comorbidities, and were considered to be non-modifiable for chronic diseases. The behavioral risks considered to be modifiable were sedentary lifestyle, smoking, alcohol consumption, overweight, diet, stress, anxiety, and unfavorable socioeconomic conditions. Environmental risks involved working conditions such as the following: number of working hours per day, week, and month; time away from home; risk of musculoskeletal injury; and opportunities for rest, hours of sleep, and access to health services. The results were presented in two categories: (1) biological, behavioral, and environmental risks, and (2) general recommendations to promote physical, cognitive, and emotional health. Macro-structural changes are needed to reorganize work and rest, improve access to health services to control modifiable risk factors, and to support behavioral and environmental changes to reduce chronic non-communicable diseases and deaths.

## 1. Introduction

The main risk factors for the development of Chronic Noncommunicable Diseases (NCD) are linked to behaviors and the environment. Risk factors include sedentary lifestyle, poor diet, smoking, alcohol consumption, and chronic stress. These contribute to the increased prevalence of HTN (Hypertension), DM (Diabetes Mellitus), obesity, smoking, PTSD (post-traumatic stress disorder), and other psychological disorders. In recent years, because of the SARS-Cov2 pandemic (COVID-19), this global NCD syndrome has been aggravated [1].

Although preventable and/or modifiable, NCDs affect two billion people, causing three quarters of the deaths worldwide, which corresponds to the death of 28 people every minute, due to some NCDs [2]. The WHO (World Health Organization) recognize that the high rate of NCDs contributes to the increase in mortality and loss of years due to disability DALYs (Disability Adjusted Life Years), and causes damage to health systems, social security, and the economies of countries [2,3].

The road transport sector moves the world economy. Without the road transport sector, and long-haul truck drivers in particular, food, supplies, medications, and various other essential products would never reach their destinations. Several studies have shown that the everyday work of long-haul truck drivers (LHTD) involves long journeys (more than 11 h daily), irregular working hours, pressure related to schedules, chronic stress, and social isolation, the absence of routine regular physical activity, and high risk of NCDs [4,5,6]. Long working hours (>55 h a week) are considered to be the largest occupational risk factor, to which around 8.9 of the population in the world is exposed [7]. The work organization of the road transport sector can negatively influence the physical, cognitive, and emotional health of workers, causing an imbalance in the work–life relationship [8].

It is important to explore the literature for the biological, behavioral, environmental, and/or occupational risk factors that influence well-being and that contribute to the development of NCDs in LHTD. Our aim is to help nurses and other professionals from the multidisciplinary health team to improve the healthcare of LHTDs after the COVID-19 pandemic. We articulated recommendations to promote the emotional, cognitive, and physical health of LHTD, to overcome the challenges to achieve the goals of the WHO’s Plan for the Prevention and Control of Chronic NCDs 2023–2030 [2], as well as the United Nations’s (UN) Sustainable Development goals [9], and present gaps in knowledge. Given this context, the following guiding question of this study was formulated: What do we know about BBERFs (biological, behavioral, and environmental risk factors) for the development of chronic NCDs in LHTD? This study aimed to analyze and synthesize the scientific findings about risk factors for the development of NCDs in LHTD.

## 2. Materials and Methods

To conduct this integrative literature review, seven steps were followed: (1) Write the review question, (2) Determine the search strategy, (3) Perform a critical appraisal of the search results, (4) Summarize the search results, (5) Perform the data extraction and reduction, (6) Perform an analysis, and (7) Consider the conclusions and implications [10].

The problem was identified, i.e., the occurrence of NCDs in LHTDs, and the following review question was established: What scientific evidence is available in the literature about BBERFs for the development of chronic NCDs in LHTD and the pandemic? The approach was based on the PICo strategy (P—Population, represented by long-haul truck drivers; I—Interest, expressed by the work–life-prevention relationship of chronic non-communicable diseases; Co—Context; Global—the COVID-19 pandemic) [11].

The selection and review of studies took place from June to December 2023 from data collection and critical evaluation of the studies. The search strategies were performed from the descriptors in Health Sciences (DECs) and the respective terms of the Medical Subject Headings (Mesh) with the Boolean operator and“Long-Haul Truck Drivers”; “COVID-19”; “Health”; “Work–Life Balance”. The databases used were the National Library of Medicine (Medline) by the Virtual Health Library (VHL), with the following intersections. On the VHL regional portal (Long-Haul Truck Driver) = 13 (Long-Haul Truck Driver) and (Work–Life Balance) = 00 (Long-Haul Truck Driver) and (Health) = 02 (Long-Haul Truck Driver) And (COVID-19) = 01 (Long-Haul Truck Driver) and (Work–Life Balance) and (COVID-19) = 00. PubMed (Long-Haul Truck Driver) = 19 and (Work–Life Balance) = 00 (Long-Haul Truck Driver) and (Health) = 00 (Long-Haul Truck Driver) and (COVID-19) = 03.

The inclusion criteria were primary human studies, with either qualitative or quantitative methodologies (or both), randomized clinical trials, prospective and retrospective cohorts, case controls, and cross-sectional studies, and were published in Portuguese, English, or Spanish between 2018 and 2022. The time period selected was based on the fact that in 2018, two important WHO documents, as follows, related to chronic diseases prevention, were completed within a decade of its publication: (1) WHO 2008–2013—Action Plan for the Global Strategy For the Prevention and Control of NCD; and (2) the Prevention and Control of NCD: Implementation of the Global Strategy [3]. Dissertations, theses, books, book chapters, editorials, newspaper articles, the literature reviews, letters to the editor, reflective studies, experience reports, and studies that did not respond to the objective of the review were excluded.

**Figure 1:**
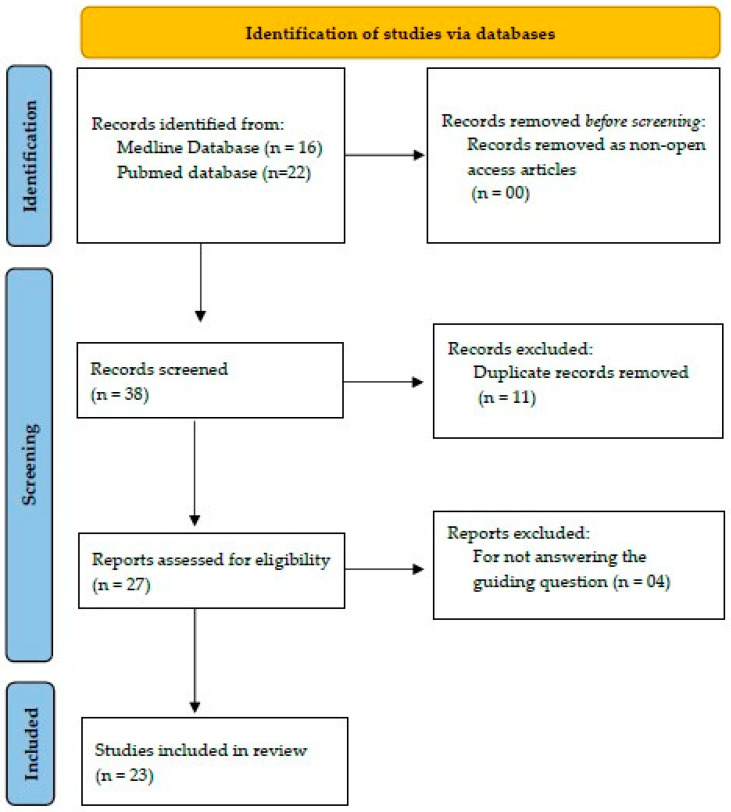
Flowchart of the process of identification, selection, and inclusion of studies. Source: Based on PRISMA for review studies [13].

The review was completed in the Rayyan application [12] and was conducted by two reviewers, and a third was consulted when questions arose. A data collection instrument was developed by the researchers, addressing the following information: authors, year, and country of publication, study subject, design, main results, and recommendations for the prevention of chronic NCDs. After the search strategies were applied, 38 studies were found (Figure 1). All titles and abstracts were read for the initial screening to determine if the studies met inclusion criteria, which resulted in the exclusion of 15 (of 38) studies. When the abstract did not clearly determine whether the article should or should not be included, the article was read in full to determine its eligibility. Twenty-three articles were fully read and were found to have met our inclusion criteria. For a summary of the search results, the guidelines of PRISMA [13] were followed.

From the strategies employed in the search and recovery, 38 studies were identified, 23 in the Pubmed database and 16 in Medline (Figure 1).

For data extraction and reduction in the selected articles, a researcher-developed instrument containing the following items was used: authorship; year of publication; objectives of the study; methodological description; characteristics of the sample; and results and conclusions.

A descriptive analysis of the data were conducted using basic information about each article. Thorough readings allowed for the convergent synthesis of themes found in the literature. To calculate the means of prevalence of diagnoses of hypertension, diabetes mellitus, cholesterol, physical activity, sedentary lifestyle, obesity, smoking habit, and alcohol consumption, a simple arithmetic mean calculation of the results was presented in the review studies, highlighting the minimum and maximum values from the studies. For the classification of biological, behavioral, and environmental domains in the presentation of risk factors for chronic diseases and general recommendations to promote physical, cognitive, and emotional health, the WHO’s prevention and control of NCDs 2023–2030 [2] document was used, and the prevention and control of NCDs—Looking Toward 2030 [3] was also used. The biological risk factors inherent to the individual, considered to be non-modifiable, were age, sex, gender, race, and chronic diseases. The behavioral and environmental risk factors, considered to be modifiable, were described as education level, physical activity, smoking habit, overweight/obesity, inadequate diet, alcohol consumption, socioeconomic conditions, occupational safety, stress, depression, anxiety, fatigue, hours of sleep, number of hours and days of work in the week and month, time out of home, ergonomic risk, and access to health services. From these analyses, it was possible to construct the conclusion and implications based on two categories, called “biological, behavioral and environmental risk factors for the development of chronic NCDs in LHTD” and “general recommendations to promote the physical, cognitive, and emotional health”.

## 3. Results

After reading the titles and abstracts, 23 articles were selected for full reading [13,14,15,16,17,18,19,20,21,22,23,24,25,26,27,28,29,30,31,32,33,34,35] (Table 1).

The studies were conducted on five continents and in 10 countries, South Africa [25,32,35], Germany [22], Australia [26,36], Brazil [21], Canada [27,30], Spain [34], United States [14,15,17,19,23,24,28,29,31,33], Finland [20], Iran [16], and Sweden [18]. In 2018, eight articles were published [14,15,16,17,18,19,20,21]; followed by six publications in 2019 [22,23,24,25,26,27], two in 2020 [28,29], five in 2021 [30,31,32,33,34], and two publications in 2022 [35,36].

The 23 studies analyzed aimed to evaluate sleep [19,20,21], sleep and mental health [23,24,29], sleep and risk of cardiovascular disease [32], diet [22], diet and physical activity [26], risk of MetS (Metabolic Syndrome) [17], cardiometabolic disease [17,18], obesity and its association with HTN and DM [35], sexual health [14], and sexually transmitted infection (STI) [33], risk of musculoskeletal injury [15], fatigue and occupational safety [34], physical and mental health [25,36], smoking [28], symptoms of depression [26], and the health of truck drivers during the COVID-19 pandemic [30].

The authors evaluated the health of 7363 LHTDs; the samples ranged from 13 to 1390 participants, with an average of 328 participants [14,15,16,17,18,19,20,21,22,23,24,25,26,27,28,29,30,31,32,33,34,35,36]. Of these, the methodology used in 19 studies was described as quantitative observational [14,15,17,19,21,22,23,24,25,26,27,28,29,31,32,33,34,35,36], a qualitative study [18], a case control study [16], and an intervention study in education [20] (Table 1). None used mixed or multiple methods.

From the analysis of the 23 articles, BBERFs that contribute to the development of chronic NCDs in LHTDs were identified (Table 2). The average prevalence of diagnoses of Hypertension was 24.6%, Diabetes Mellitus (11.6%), High Cholesterol (27.2%), Practice of Physical Activity (38%), Sedentarism (38.8%), BMI > 25 (55.3%), Obesity (48.4%), Smoking habit (37.1%), and Alcohol consumption (48.5%).

The general recommendations to promote physical, cognitive, and emotional health are presented based on the themes addressed in the studies and are described following the recommendations of the studies, and the WHO documents [2,3] as a way to promote the health of LHTDs are described in (Table 3).

## 4. Discussion

When analyzing the articles regarding the evaluation of the occurrence of chronic NCDs and the recommendations of studies for the promotion of health of LHTDs, the results was presented in two categories: (1) biological, behavioral, and environmental risks; and (2) general recommendations to promote physical, cognitive, and emotional health.

### 4.1. Biological, Behavioral, and Environmental Risk Factors for the Development of Chronic NCDs in LHTD

From the evaluation of the studies, it was possible to identify BBERFs for the development of chronic NCDs in LHTDs. Among the biological factors, not modifiable for the development of chronic NCDs, the studies presented data on age, race, genetics, and diagnosis of chronic diseases. Regarding age, 55% and 60% of the studies showed that LHTDs were over 45 years old [17,36]; other studies found an average of more than 46 years old [19,30,35], 50 years [24,27], and 53 years old [14,15,21,22]. Male sex is the most affected by premature deaths due to chronic NCDs [37], and in all studies analyzed there was a predominance of male participants [14,15,17,18,21,22,25,27,28,30,31,32,34,36]. Only one study had a predominately female sample [29]. African Americans presented a higher risk for developing diabetes mellitus, although the sample in the articles were predominately Caucasian participants [14,15,19,27,29]. The study developed in South Africa had a predominance of African people in their sample [32].

Some chronic diseases have an important genetic component for health, such as HTN, diabetes mellitus, and obesity. The description of risk of diseases related to genetic factors was pointed out by some studies. A qualitative study pointed the cause of death of the driver’s father was due to heart attack, 3% of the participants revealed that their parents had stroke before the age of 60 [25], and 5% had a history of acute myocardial infarction [14]. In other studies, the description of the diagnosis of some chronic non-communicable disease was frequent in 51% of LHTDs, with at least one chronic disease, and there was a higher prevalence for arterial hypertension (33%) and high cholesterol (28%) among participants [23]. In total, 39.8% had high blood pressure diagnosis, 45.8% had high cholesterol, and 14% had diabetes mellitus [14]. In total, 71% of those who were overweight had some chronic disease (hypertension, diabetes, and or chronic back pain) [22,30], and 30% reported at least three chronic diseases [36]. For the diagnosis of hypertension, a mean of 22.5% was identified, with a minimum of 11% [25] and a maximum of 34% of hypertensive drivers [36]. Diabetes mellitus was identified on average in 14.5% of drivers, ranging from 2% [25] to 27% [17], and a mean of 27.2%. The diagnosis of high cholesterol varied from 7.8% [36] and 45% [14] of the participants of the studies.

Education level is a determining factor in health due to increased exposure to risk factors and limitations in the access to information and health services. The predominant level of education among drivers was high school in 91% [22], followed by 56% who studied between four and eight years [21]. In total, 26% completed high school and more than 20% had studied at college [30].

The development of chronic diseases is related to sedentary behavior, and the evaluation of physical activity showed that, on average, 30.5% practiced physical activity three hours a week or more [31], with variation from 1% to 60% [23]. Similarly, the average of sedentary drivers was 48.5%, ranging from 26% to 45% [32,35].

Healthy eating is a basic human right, promotes health, and is related to lower chronic disease risk. Regarding the diet of LHTDs, 74.2% reported spending less than an hour a day in the preparation of meal and eating [23], 73% reported eating food brought from home, and 32% reported eating the main meal in the places of stopping and resting [22], 88% consumed less vegetables than recommended, 63% at least one health-damaging food per day, and two thirds consumed a can of high-sugar drink daily [26]. In addition, the difficulty of finding restaurants that were open during the COVID-19 pandemic was reported by 82% of drivers [30]. The control of weight is considered fundamental for reducing the prevalence of chronic diseases. Among the studies evaluated, overweight was identified in studies that considered BMI > 25, the mean was 55.3%, with a minimum variation of 25.2% [32,36] and 90% [26], and obesity was, on average 48.4%, ranging from 28% [22,32,35] to 69% [25]. Significant correlation was identified among drivers who ate meals in restaurants with weight gain during the COVID-19 pandemic [30].

Among the modifiable behavioral risk factors for NCD illness, studies showed that tobacco consumption by LHTDs was on average 37.1%, with variation between 1.8% [21] and 68% [28]. Among smokers, 48.8% already attempted to cease the smoking behavior at least once in the previous year [14]. The use of alcohol on days off work for 48% [23], and 50% reported consumption in the last 30 days [14]. Other studies have shown that the average usual consumption was 48.5%, with variation between 39% [21] and 57% [35]. Alcohol consumption by drivers compromises safety, and as well as tobacco use, increases the risk of chronic diseases, and requires changes in behavior and prevention and control policies to reduce the consumption and marketing of cigarettes and alcohol harmful to health.

Income is part of the socioeconomic conditions which define the living and working conditions of people. In the evaluation of drivers’ income, 54% earned more than USD 80,000 per year, with 95% of their revenue going to truck maintenance and labor-related expenses [14].

Increased stress at work may increase depression rate among drivers (HATAMI et al., 218). In a study of anxiety, depression, sleep, and fatigue, the researchers evidenced an association between depression and work; drivers without co-pilots presented moderate to severe depression, while those with co-pilots had lower rates of depression, compared to those who worked alone [16]; 7% had anxiety and 12% had depression [19], 40% had moderate perceived stress [24]; 8% had Depression, 4% had Post-Traumatic Stress Disorder, and 18% had Daytime Drowsiness [25]; 19.4% had mental health problems [36]; 9% had severe headache and 10% had chronic fatigue, 60% had moderate to chronic stress [23], 62% had moderate or high perceived stress [24], and 10% reported symptoms of anxiety or loneliness [29]. Drivers who reported depressive symptoms had a higher chance of lower-back pain, fatigue, fewer hours of sleep per night, and greater use of medications [27], and 85% of drivers with depressive symptoms did not maintain follow-up appointments with their healthcare providers [27].

Time spent at work can be a barrier to changing behavior and adopting healthy habits. The number of work days per week and month were described as more than five days per week in 73% [34], and as more than 15 days per month by 85% [22,24], and an average of 20 days/month [25], more than 25 days per month, in 84% of participants [24]. As for working hours per day, the classification was five to eight hours per day in 11% and 53% [34,36], 30% worked daily more than eight hours [21], and 53% more than 10 h [32]; the mean was 10 h per day [25], while more than 11 h per day was reported by 32% [24] and 50.4% [36], and more than 13 h per day by 37.5% [36] and 38% [24]. It is understood that during the COVID-19 pandemic, the workday for drivers was extended, considering that drivers carried essential items such as food and medical hospital materials, which led to 58% of drivers to report loss in well-being from the outset of the COVID-19 pandemic [30].

Sleep hygiene is essential for the prevention of chronic diseases such as HTN, diabetes mellitus, obesity, depression, and road accidents. However, 29% of long-haul truck drivers reported worsening sleep quality during the COVID-19 pandemic [30]. Sleeping fewer than six hours compromises health, but it is a modifiable risk factor, and the evaluation of the sleeping hours was on average less than seven hours for 46% of drivers [23], seven [19], and more than seven [21,29,32]. The amount of time out of the house (i.e., on the road, working) and the feeling of loneliness varied according to the relationship with the company; independent truck drivers stayed out of the house for significantly more nights than company truck drivers [14], and 76% reported frequent support from supervisors [24]. In general, the period far from home varied in more than 21 days to 84% [24], and more than 14 days to 33% [22].

The driver’s work involves ongoing monotonous activity for hours, and this can compromise safety. In order to maintain social interaction and decrease boredom, drivers performed secondary tasks while driving [18] and reported that they maintain social interaction and communication with friends and colleagues by smartphone and virtual social networks and in places of stopping and rest [18].

The working conditions of drivers can cause acute musculoskeletal injuries that can then become chronic. The risk assessment of musculoskeletal injury showed that for 53% of drivers, the injuries led to the removal of work in the last 12 months [15]. Of these injuries, 30% were the result of a drop in the same level and 32% were by contact with objects and or equipment, and 15% were by effort [15]. The injuries occurred to the arm, shoulder, and hand for 26% of drivers, and occurred in the neck and back for 21%, and 15% had injuries occurring in the legs [15]. In other studies, 27% work-related injury and 26% reported chronic back pain [22]; 10% had work-related pain that lasted more than three months [25], and 40% had chronic pain lasting 3 to 12 months [36], demonstrating the potential for chronicity.

The difficulty in accessing health services by LHTDs compromises the prevention of chronic NCDs. In an evaluation of health monitoring by professionals, 32% reported monitoring annually or every two years to 62.8%; 32% of truck drivers reported conflicts between work schedules and hours of care in health services, and 19.6% cited financial problems that prevented them from obtaining health care [14]; 85% of the LHTD that expressed symptoms related to depression did not receive psychiatric medications, and did not receive care from mental health professionals (80%) [27].

Given the evidence of impacts on food in the face of the difficulty of finding open restaurants during the pandemic, the correlation of weight gain between those who dined in restaurants during the pandemic, increased work demand, and loss of well-being and worsening quality of life, sleep, and difficulty finding a vacancy to park [30], it can be said that the pandemic contributed to aggravating risk factors related to the development of chronic NCDs in LHTD and in worsening the health results of healthy LHTDs.

### 4.2. General Recommendations to Promote Physical, Cognitive, and Emotional Health in LHTD

The general recommendations of the studies to promote physical, cognitive, and emotional health are presented as a way to promote the health of LHTDs and are described in Table 3. These are categorized into behavioral and environmental areas (modifiable). In the behavioral area, the recommendations from this review about the promotion for physical activity are presented to increase the opportunities of physical activity with the installation of gyms in the places of stop and rest, availability of hiking trails and physical activity with supervision of health professionals, as well as providing health education on the importance of physical activity [14,17,23,31,32].

As for healthy eating, the need to provide health education for changing behavior was highlighted, increasing the opportunities for healthy diet, with the availability of nutritious foods with fruits, vegetables, nutritious meals free of excess salt, saturated fats, trans fats, and sugars [14,17,22], as well as the recommendation for the automotive industry to improve food preparation sites in trucks [22]. It must be considered that 56% reported that finding a place to park is always a problem, and for 64% it was even higher during the COVID-19 pandemic [30].

The promotion of sleep and rest health was addressed in recommendations on the importance of encouraging rest and sleep hours [17,19,27,32], highlighting the importance of ensuring that the place of sleep is quiet, with adequate temperature control, and with quality air [17]. Also, to provide health education on the importance of sleep quality [27]; to monitor, detect, and offer information on sleep changes and association with the risk of cardiovascular diseases [19], use new technologies to enable personalized, intensive, and relevant training [20,32], and use strategies such as physical exercise, avoiding caffeine or other stimulants before bedtime [17], as well as providing resources and incentives to manage weight and reduce the risk of sleep disorders [17] and implement sleep disorder screening and treatment programs [17]. Still in relation to sleep quality, a study showed that when monitoring the use of drugs, 53% of drivers who used antihypertensive drugs reported good or optimal sleep quality [21].

In addition, there is a need for initiatives to prevent STIs with interventions to evaluate behavior and health education to prevent and control risk of infection by STI/HIV [32,33]. Health education interventions for smoking prevention and control [14], as well as text message interventions three times a week and counseling from 10 to 15 min [28]. Similarly, the recommendations for health education interventions are recommended for the prevention and control of the use of psychoactive/narcotic substances/drugs/alcohol [27], with an indication of mental health professional attendance via telephone, within 24 h (tele-health), or in online support groups, the availability of emotional support and stress management techniques [24,27,29], occupational stress level assessments [17]. In addition, one can explore the viability, acceptability, and effectiveness of Mindfulness practice. To encourage the practice of Mindfulness, considering that the practice of Mindfulness acted as a protector against Post-Traumatic Stress Disorder [29].

In the environmental area, recommendations involving the work environment were included. These were described as general recommendations and highlighted the importance of expanding the opportunities for stopping and rest [14,23,34], with availability of space for basic needs and meals with tables and chairs [14,22].

For the prevention of injuries, it is recommended to evaluate and control risk factors and the mechanism of injuries and use evidence to develop prevention and intervention protocols in work-related injuries [15]. The reorganization of the working day was pointed out as urgent, considering that more than 80% reported having a different daily work schedule each day [24] and the reorganization of working hours can act in the prevention of strenuous working hours [34], considering that professionals who had extensive journeys had four times more chances of depression [27]. On the other hand, drivers with co-pilots had lower stress and depression rates [16].

There is an urgent recommendation to promote access to health care services for the prevention of NCDs, control of hypertension, diabetes mellitus, and obesity, as well as the medications necessary to control non-communicable diseases. Healthcare services, which could be face-to-face at health centers at truck stops or close to roadways or online via telehealth, need to include physical assessment, blood pressure, and weight monitoring, but also detailed sleep assessments because of their association with the risk of metabolic and cardiovascular diseases [14,27,31,32].

From the studies analyzed in the review, there seems to be little difference among the risk factors for the development of chronic LHTD diseases between countries and/or continents, possibly due to biological and or cultural characteristics. On the other hand, general recommendations for promoting physical, cognitive, and emotional health can be adapted to different cultures and health systems.

Faced with the feeling of loneliness and the need for a social support network [14], the role of the work organization has the potential to improve work–life relationships [8], as long as it promotes personal growth opportunities capable of influencing choices and healthy behaviors, promoting changes in physical, cognitive, and emotional health. The gaps identified in the literature addressed two important issues for the prevention of chronic diseases. The first gap is knowledge about the social support network in and outside the workplace. Improving opportunities for social interaction in places of rest and rest with spaces for sports activities and intellectual games could have the potential to improve physical, cognitive, and emotional health. Social interaction improves one’s sense of belonging, favors resilience, increases concentration, and reduces loneliness and stress. The second gap is the absence of studies addressing opportunities for personal growth, learning spaces, such as libraries, face-to-face or online training, that promote the development of skills and individual growth in the workplace, in places of rest, or remotely. Encouraging self-development, continuing education, skills development, and participation in training can contribute to improving cognitive and emotional health. In addition, these activities can improve memory, performance at work, problem-solving, concentration, interpretation, and health literacy, and promote informed decision-making in health for the prevention and control of chronic NCDs.

### 4.3. Limitations

The possible limitations of this study may be related to the scarcity of studies produced during the COVID-19 pandemic period, which did not allow the deepening of how risk factors changed or were accentuated due to the need for care to prevent contagion. In addition, the methodological strategy adopted may have influenced the result, since it is estimated that the increase in search time and the inclusion of other databases and other languages could have expanded the sample. However, it is considered that this does not decrease the quality of the study, considering that appropriate methodological strategies were adopted to obtain the best scientific evidence, developed by researchers recognized in their areas, and published in scientific journals with high-impact indexes.

### 4.4. Contributions to Practice and Future Studies

The evidence raised in this study can contribute to improving the approach of professionals to the health of LHTDs, in the prevention of chronic diseases, as well as to promote reflection on the need for urgent changes in legislation and work organizations, as well as in the environments of places of rest so that measures aimed at the prevention of chronic NCDs are implemented. In addition, gaps were found in the scientific literature related to the absence of studies that addressed the influence of a social support network and the encouragement of personal growth in health promotion, and which encouraged the development of other studies that promoted advances in the prevention and control of chronic NCDs present in this population.

## 5. Conclusions

The results of this study reported a synthesis of the evidence about the risk factors for the development of chronic NCDs in LHTDs and described recommendations for the promotion of physical, cognitive, and emotional health of LHTD. As indicated by the data, it is clear that macrostructural changes are needed which may improve working conditions, from the work and rest environment of LHTDs, and may reduce barriers and allow for behavioral changes such as increasing access to health services, increasing medications for control and treatment of NCDs, increasing health education, and implementing programs to encourage physical activity, healthy eating, and reductions in sedentary behavior, obesity, stress and depression.

The general recommendations of the studies to promote the physical, cognitive, and emotional health include the promotion of physical activity practice, healthy eating, and health education on the quality and importance of sleep hours, the control of tobacco and drug use, the dangers of STIs, and the benefits of work reorganization, considering that long journeys are considered the highest occupational risk for heart disease, stroke, and deaths. For this, the demand to promote an environment conducive to changing behavior needs to be part of the global NCD prevention agenda. The authors recommended that future research be conducted to evaluate strategies to strengthen the social support network and opportunities for personal growth in the prevention of chronic diseases and health promotion of LHTDs in the post-COVID-19 pandemic period.

## Figures and Tables

**Table 1:** Characterization of health assessment studies of long-haul truck drivers published between 2018 and 2022.

Author/Year Country Type of Study Number of Subjects Study Subject	Main Results
Bachman et al., 2018 [14]. USA Observational Study *N* = 266 Sexual health of LHTD in the US.	In total, 17.3% assumed to have had at least one STI in the previous year, the most frequent being gonorrhea (63.6%). The average of HIV/AIDS infection was lower than the national average in the US. However, cholesterol, diabetes, obesity, and smoking were higher than the average in the US.
Hcombs et al., 2018 [15]. USA Observational Study *N* = 1265 Work-related musculoskeletal injuries in LHTD.	The majority of lesions were on the arm (26.3%) and back (21.1%). Falls were caused by objects or equipment (33.7%), resulting commonly in sprains (60%), and were responsible for causing musculoskeletal lesions (38.9%).
Hatami et al., 2018 [16]. Iran Experimental study *N* = 70 drivers (33 drivers with co-pilot—intervention group; 37 drivers without co-pilot—control group). Effect of co-pilots on mental health in LHTD.	The proportion of LHTD with depression was lower in those who work with a co-pilot.
Hege et al., 2018 [17]. USA Observational Study *N* = 115 The characteristics of the work organization experienced by LHTDs and risks of cardiometabolic diseases.	In total, 84.6% reported staying on the road for more than three weeks per month. In total, 82.7% do not have a fixed work schedule, which varies daily. In total, 77.7% expressed feeling pressured in relation to time, 70% worked more than 11 h daily, 68% described their work routine as rushed, and 62.5% had moderate to chronic stress. Longer sleep duration on working days (average 8.27 on working days vs. 6.95 h on business days). In total, there was an 11% diagnosis of sleep apnea.
Iseland et al., 2018 [18]. Sweden Observational Study *N* = 13 The secondary tasks while driving.	Regarding the secondary tasks, the interaction with the cell phone were mainly for communication, entertainment (social media and music), and GPS. They referred to performing them to reduce boredom and stress.
Lemke et al., 2018 [19]. USA Observational Study *N* = 260 Sleep disorders.	Risk factors corresponded to known symptoms and were related to safety and health. The five main risk factors for sleep disorder were related to the following: Circadian rhythm; Respiratory; Parasomomia; Insomnia; and SRMD.
Pylkkönen et al., 2018 [20]. Finland Observational Study *N* = 53 Educational intervention on the drowsiness of LHTD.	The results of multilevel regression models did not show significant improvements related to intervention in driver drowsiness in the night or morning shift compared to day or night workers.
Rodrigues et al., 2018 [21]. Brazil Observational Study *N* = 367 The socio-demographic profile, the working day, and the general health conditions of freight transport professionals on highways with the reported sleep regime.	Regarding the prevalence of overweight men, 26% reported being hypertensive and 9% diabetic, 32% used drugs, 30% worked more than eight hours a day, 18% were smokers, and 39% consumed alcohol.
Bschaden et al., 2019 [22]. Germany Observational Study *N* = 404 Patterns of food choice of LHTD.	In total, 24% had average weight, 46% were overweight, and 30% were obese with at least one chronic disease. More than 50% reported being a smoker. In total, 37% usually or always had meals at truck stops, and 6% did not have the habit of eating in these places. In total, 73% reported eating food brought from home. These items were sandwiches (38.7%), sausages (50.6%), raw vegetables (31%), sweets (35.4%), fruits (62%), and pre-ready meals (37%). In total, 94% had these items in the truck refrigerator, 62% had them in the gas pot, and 8% had them in the microwave, and ate less often in the places in which they stopped and rested.
Hege; Apostolopoulos; Sönmez, 2019 [23]. USA Observational Study *N* = 260 Connections between the organization of long-haul truck driver work, stress at work, sleep, and health.	More than 70% reported working more than 11 h a day, with 46% sleeping less than seven hours daily. This resulted in higher chances of high stress at work, caffeine consumption, and a decrease in the quality of sleep. In total, 48% consumed alcohol on non-work days, and 48% were smokers.
Hege et al., 2019 [24]. USA Observational Study *N* = 260 Stress and sleep.	The perceived stress was related to work hours and quality of sleep.
Lalla-Edward et al., 2019 [25]. South Africa Observational Study *N* = 614 Health information of LHTD in South Africa.	More than 85% reported having sexual relations with regular partners, and more than 25% reported having sex with casual partners; 14% had sex with a sex worker. In total, 50% never worked in the night shift, and 12% worked nights approximately four times a week. In total, 8% were HIV positive, with half taking antiretrovirals.
Sendal et al., 2019 [26]. Australia Observational Study *N* = 231 Self-reported behavior of diet and physical activity.	In total, 85% worked more than nine hours a day. Half consumed fruits, and almost 90% consumed vegetables beneath the national recommendations. More than two-thirds reported consuming at least one health-harmful food and a sugary drink. Two thirds were obese, and 90% had low muscle mass index.
Crizzle; Malkin, 2020 [27]. Canada Observational Study *N* = 107 Identify predictors of depressive symptoms.	In total, 95% of the participants were male, with 44% reported having presented depressive symptoms in the last year. The results suggest that occupational stressors contribute to increasing the risk of depressive symptoms in the worker. The results showed the association between time dedicated to work, annual income, and depressive symptoms.
Kagabo et al., 2020 [28]. USA Observational Study *N* = 37 Smoking and their preferred methods of smoking cessation among LHTD.	In total, 68.8% were regular smokers, using more than 15 cigarettes daily. The reasons for smoking behavior was staying attentive, reducing stress, or having something to do while driving. In total, 65% made at least one attempt to stop.
Wise; Heaton; Shattell, 2020 [29]. USA Observational Study *N* = 140 Sleep, mental health, health care use, and mindfulness.	In total, 70% of the participants were female, 90% Caucasian, mean 37 years of age. In total, 14% presented symptoms of depression, with 10% presenting symptoms of anxiety or loneliness. Symptoms of PTSD and daytime somnolence were identified. Mindfulness was inversely correlated with the symptomatology of Post-Traumatic Stress Disorder.
Crizzle; Malik; Toxopeus, 2021 [30]. Canada Observational Study *N* = 146 Working conditions preceding and throughout the pandemic period regarding access to food, bathrooms, and parking.	The access to food, bathrooms, and parking during the COVID-19 pandemic was reported by the LHTD as problematic during this period. Also, they worked more, consumed more caffeine, and half reported being tired.
Lemke et al., 2021 [31]. USA Observational Study *N* = 115 insulin sensitivity.	Most of the interviewees, 47.6%, were white and had a diagnosis of diabetes. In total 13% used diabetes medicines, and 67% were obese. The average insulin concentration was higher among truck drivers, but the average glucose concentrations were lower among truck drivers compared to among NHANES participants.
Roche et al., 2021 [32]. South Africa Observational Study *N* = 575 Sleep disorders and risk of cardiovascular diseases.	Mean age of 37 years. In total, 17% were at risk of OSA, and 72.0% had high blood pressure. Almost 50% reported working overnight at least once every 7 days. Almost a third of participants were obese. Sleep duration was an average of 7 h. In total, 9.4% had HIV.
Patterson et al., 2021 [33]. USA Observational Study *N* = 88 (58 male truck drivers, 24 sex professionals, and 6 male intermediaries). Sexuality.	In total, 27% tested positive for STI/HIV or hepatitis. People who tested negative for an infection involved in sex and/or drug exchanges with people who tested positive, increasing their risk of infection/transmission to other contacts.
Useche et al., 2021 [34]. Spain Observational Study *N* = 521 Fatigue, work stress, health indicators, and occupational traffic accidents.	In total, 47.9% were male, and 53% worked five to eight hours a day. The workplace accidents of LHTDs may be related to fatigue caused by work.
Modjadji et al., 2022 [35]. South Africa Observational Study *N* = 96 Obesity, HTN, and DM.	Almost one third of the sample showed obesity, 44% were overweight, and 57% had abdominal obesity and 14% were diabetic.
Van Vreden et al., 2022 [36]. Australia Observational Study *N* = 1390 Physical and mental health.	Most drivers were obese. Almost 30% had at least three chronic diseases. The main problem reported was in relation to the spine, followed by HTN and mental health.

**Table 2:** Biological, behavioral, and environmental risk factors for the development of NCDs in LHTDs.

Domain	Risk Factor	Detail	References
Biological (non-modifiable)	Age	(>45 years)	[14,15,17,19,21,22,23,27,30,35,36]
	Sex	Male	[14,15,17,18,21,22,25,27,28,30,31,32,34,36]
		Female	[29]
	Race	Caucasian	[14,15,19,27,29,30,31]
		Afro-descendants	[32]
	Chronic disease	DM HTN MetS	[14,17,19,21,22,25,30,36]
Behavioral and/or environmental (modifiable)	Education	Up to 8 years	[21,27]
		>8 years	[14,22,30]
	Physical activity	Sedentary	[17,23,25,31,32,35]
	Habit of smoking	Smoking	[14,21,22,23,25,28,30,35]
	Alcohol consumption	Working days/ Days off	[17,21,27,30,35]
	Overweight/Obesity		[14,17,21,22,25,26,27,30,32,35,36]
	Diet	Food	[22,23,26]
	Socioeconomic conditions	Salary	[14,30]
	Mental health	Anxiety/stress/depression/sleep and fatigue	[16,17,19,23,25,27,29,30,36]
	Occupational safety		[18]
	Working days per week/month		[22,23,25,30,34]
	Working hours per day		[21,23,25,32,34,36]
	Hours of sleep		[21,29,30,32]
	Time out of the house	Loneliness	[14,18,22,23]
	Ergonomic risk	Skeletal muscle injury	[15,22,25,36]
	Access to health services		[14,27]

**Table 3:** General recommendations to promote the physical, cognitive, and emotional health of LHTD.

Domain	Recommendation	Justification	Reference
Biological	Prevent and control hypertension, diabetes mellitus and obesity	Monitor height, weight, and abdominal circumference and body mass index. Supporting initiatives promoting health education, physical activity, healthy diet, opportunities for rest, sleep, medicines, and health services, as well as reducing stress, smoking, and alcohol consumption, contributes to reducing morbidity and mortality by NCD, and promotes physical and mental health.	[14,23,31,32]
Behavioral	Promote regular physical activity	It reduces the risk of diseases such as obesity, DM, HTN, OSA, stress, fatigue, pain, and anxiety. Increases safety and improves concentration and sleep.	[14,17,23,31,32]
	Provide healthy eating	Decreases salt intake, and saturated trans-fat contributes to reduce overweight and risk of cardiometabolic diseases. Improves memory and increases well-being.	[14,17,22]
	Provide adequate amount of sleep hours	Health education on the importance of maintaining circadian rhythm with up to eight hours of sleep and implementing measures to respect sleep and rest time can favor the prevention of cardiovascular diseases, decreases HTN, diabetes mellitus, obesity, pain, memory loss, stress, and risk of depressive symptoms. Increases concentration and reaction.	[17,19,20,27,32]
	Control smoking	Decreases the risk of developing cardiometabolic disease, brain injuries, and improves sleep. Increases the feeling of pleasure and reward.	[14,28]
	Control alcohol/drug consumption	It reduces the risk of cardiovascular diseases, increases brain activity, and favors the optimization of the sleep–wake cycle and reduces emotional problems.	[27]
	Promote mental health	Promotes improvement of emotional control and cognitive and physical performance. Encouraging the practice of Mindfulness increases concentration and occupational safety and prevents stress, symptoms of PTSD, and feelings of anxiety and loneliness.	[17,23,29,31]
	Prevent Sexually Transmitted Infection (STI)	Providing health education to reduce the risk of sexually transmitted infection, HIV/AIDS, and cancer.	[14,32,33]
Environmental	Promote rest with the expansion of the places of stop and rest	Decreases the risk of fatigue, pain, accidents, drowsiness, stress, symptoms of depression, and overweight. Increases safety at work and sleep quality. Prevention of the risk of cardiometabolic diseases, obesity, and cancer. Reduces social isolation and stress, and increases well-being.	[14,22,23,34]
	Health education to prevent mechanical injuries	Promotes health, more effective management of injuries, and the appropriate development of the care plan.	[15]
	Reorganize the work day	Sharing responsibilities with a co-pilot colleague decreases the driver’s loneliness, promotes free time to plan the achievement of new goals, and increases job satisfaction and well-being. Lower number of hours at the wheel contributes to the reduction in cardiometabolic diseases, increases occupational safety, and reduces the level of stress and depression. Reducing long journeys increases the time available for family life, and reduces the risk of illness and death.	[16,27,34]
	Promote access to health care services	It prevents chronic diseases, increases productivity, prevents depressive symptoms, and improves quality of life.	[14,19,27,32]
	Promote access to essential medications for the control of NCD	Decreases the risk of premature death and increases occupational safety, concentration, and reduce mood swings.	[21,31]

## Abbreviations

Abbreviation	Meaning
BBERFs	Biological, Behavioral, and Environmental Risk Factors
DALYs	Disability Adjusted Life Years
DECs	Descriptors in Health Sciences
DM	Diabetes Mellitus
GPS	Global Positioning System
HTN	HTN
HIV	Human Immunodeficiency Virus
LHTD	Long-Haul Truck Drivers
MetS	Metabolic syndrome
NCD	Chronic Noncommunicable Diseases
NHANES	National Health and Nutrition Examination Survey
OSA	Obstructive Sleep Apnea
PTSD	Post-Traumatic Stress Disorder
PRISMA	Preferred Items of Systematic Reviews and Meta-Analyses
SRMD	Sleep-Related Movement Disorders
STD	Sexually Transmitted Disease
STI	Sexually Transmitted Infection
UN	United Nations
US	United States
WHO	World Health Organization
